# Grid multi-category response logistic models

**DOI:** 10.1186/s12911-015-0133-y

**Published:** 2015-02-18

**Authors:** Yuan Wu, Xiaoqian Jiang, Shuang Wang, Wenchao Jiang, Pinghao Li, Lucila Ohno-Machado

**Affiliations:** 1 Department of Biostatistics & Bioinformatics, Duke University, Durham, NC 27708 USA; 2 Division of Biomedical Informatics, Department of Medicine, University of California, San Diego, La Jolla, CA 92093 USA; 3 Department of Electronic Engineering, Shanghai Jiaotong University, Shanghai, 200240 China

**Keywords:** Grid MLE, Ordinal logistic model, Multinomial logistic model

## Abstract

**Background:**

Multi-category response models are very important complements to binary logistic models in medical decision-making. Decomposing model construction by aggregating computation developed at different sites is necessary when data cannot be moved outside institutions due to privacy or other concerns. Such decomposition makes it possible to conduct grid computing to protect the privacy of individual observations.

**Methods:**

This paper proposes two grid multi-category response models for ordinal and multinomial logistic regressions. Grid computation to test model assumptions is also developed for these two types of models. In addition, we present grid methods for goodness-of-fit assessment and for classification performance evaluation.

**Results:**

Simulation results show that the grid models produce the same results as those obtained from corresponding centralized models, demonstrating that it is possible to build models using multi-center data without losing accuracy or transmitting observation-level data. Two real data sets are used to evaluate the performance of our proposed grid models.

**Conclusions:**

The grid fitting method offers a practical solution for resolving privacy and other issues caused by pooling all data in a central site. The proposed method is applicable for various likelihood estimation problems, including other generalized linear models.

**Electronic supplementary material:**

The online version of this article (doi:10.1186/s12911-015-0133-y) contains supplementary material, which is available to authorized users.

## Background

In biomedical research, data sharing plays an important role in accelerating scientific discoveries. For example, networks based on information from electronic health record (EHR) [1,2] have been established for this purpose. However, due to privacy concerns, patient-level data cannot always be exchanged across different institutions. In these circumstances grid computing, which avoids sharing patient level data among multiple institutions, can be used to build a global model.

For example, logistic regression models have been used in a variety of clinical applications, such as scoring candidates for liver transplant using the Model for End-stage Liver Disease [3], producing estimates related to myocardial infarction diagnosis [4], and detecting suspicious accesses to electronic health records [5]. These scenarios, in their classical setups, have difficulties in handling multi-center data, as the training phase requires accessing the entire dataset.

Our previous work [6] and [7] proposed privacy-preserving models through the aggregation of non-sensitive intermediary results (i.e., gradient and Hessian matrix for the log-likelihood function), but the model only deals with binary models. Response variables with more than two categorical values occur very often in medical models. For example, cancer progress is often categorized into 4 or 5 phases. One simple method to deal with multiple responses is to fit binary logistic fitting for each pair of these multiple categories. However, this approach is very inconvenient and the performance of each binary logistic model might be degraded when sample size is insufficient. Some researchers extended the binary logistic model to handle multi-category response problems. Among existing approaches, ordinal logistic [8] and multinomial logistic [9] are the two most popular multi-category response logistic models for ordinal and nominal responses, respectively. Both methods are widely used to fit data with multi-category response. However, methods for binary model fitting assessment may not be applicable to multi-category problems. Hosmer and Lemeshow [9] introduced novel methods to evaluate the goodness-of-fit of multi-category logistic models. The Area Under the ROC Curve (AUC) [10] is an important measure in checking classification performance of binary outcome models. Hand and Till [11] generalized the original AUC measure to deal with classification methods for multi-category outcome cases. The AUC for binary logistic regression is given by

$$ \widehat{A}\left(1\Big|0\right)=\frac{R-\left[\left({n}_1+1\right){n}_1\right]/2}{n_1{n}_2}, $$

where *n*_1_, *n*_2_ are the number of observations with *Y*=1 and with *Y*=0, *R* is the rank sum based on the predicted probability of *Y* = 1 for observations with *Y* = 1 among all observations. Van Calster *et. al.* [12] described several AUC score estimation methods for the ordinal logistic model, one of which is to use the mean of AUC scores from *K* − 1 binary logistic regression estimations to serve as the AUC score for ordinal logistic model. Hand and Till [11] defined *Â*(*k*_1_|*k*_2_) in the same way for observations with *Y* ∈ {*k*_1_, *k*_2_} and proposed a generalized AUC for multinomial logistic model as $\frac{2}{K\left(K-1\right)}{\displaystyle {\sum}_{k_1<{k}_2}}\ \left[\widehat{A}\left({k}_1\Big|{k}_2\right)+\widehat{A}\left({k}_1\Big|{k}_2\right)\right]/2$ for 1 ≤ *k*_1_, *k*_2_ ≤ *K*. Yang and Carlin [13] generalized the ROC curve to a surface and used the volume under the ROC surface (VUC) to measure the accuracy of a diagnostic test based on multi-category response models. Dreiseitl *et. al.* [14] proposed to use a three-way ROC curve analysis for the same goal. Van Calster *et. al.* [12] suggested an ordinal c-index measurement (ORC) and discussed the relationship between the new measurement with VUC and other measurements based on assessing pairs of cases.

In this article, we introduce grid ordinal and multinomial logistic models to handle multi-center modeling of multi-category response, including model assumption checking. We also propose to use the grid AUC score to evaluate the added value of the grid model fitting when compared to models fitted by separate sub-datasets. The remainder of this article is organized as follows. The second Section briefly reviews ordinal logistic [8] and multinomial logistic [9] models and their model assumptions, and also discusses model coefficient estimation methods for both models and the statistical test for checking the ordinal logistic assumption. The third Section discusses grid maximum likelihood estimation and grid computing for the ordinal logistic model assumption test statistics. The fourth Section provides technical details for grid model fitting assessment. The fifth Section elaborates on grid AUC score computing. The sixth Section describes simulation studies to evaluate the theoretical results. The seventh Section carries out additional experiments on two real datasets to demonstrate our proposed methods. The eighth Section discusses the generalization of the proposed grid models and the limitations of this work.

## Methods

### Ordinal and multinomial logistic models

Before we introduce our method we first introduce both ordinal and multinomial logistic models in a few more detail. In terms of how to split response categories, many ordinal logistic models have been studied. However, in this article, we only focus on the proportional odds logistic model to deal with multi-category problems. The proposed method will be extended to other multi-category logistic regression models in the future. Suppose response *Y* could take values 1, ⋯, *K* (for *K* categories) with K ≥ 3. There are *m* features in the model and *n* observations. The predictor matrix can be expressed as *X*^*T*^ = (*x*_1_, ⋯, *x*_*n*_) with ${x}_i^T=\left({x}_{i,1},\cdots, {x}_{i,m}\right)$ for 1 ≤ *i* ≤ *n*. Let’s define *p*(*w*, *i*) ≜ *Pr*(*Y* ≤ *w*|*x*_*i*_) and assume 1 ≤ *i* ≤ *n* and 1 ≤ *w* ≤ *K* − 1. The ordinal logistic regression [8] can be defined as

1 $$ p\left(w,i\right)=\frac{e^{\alpha_w+{\beta}^T{x}_i}}{1+{e}^{\alpha_w+{\beta}^T{x}_i}}, $$

With parameters *β*^*T*^ = (*b*_1_, ⋯, *b*_*m*_). The conditional likelihood function is given by

2 $$ \begin{array}{l}L={\varPi}_{i=1}^n\left\{p{\left(1,i\right)}^{I_{\left[{y}_i=1\right]}}{\varPi}_{w=2}^{K-1}{\left[p\left(w,i\right)-p\left(w-1,i\right)\right]}^{I_{\left[{y}_i=w\right]}}\right.\\ {}\kern2.75em \left.\cdot {\left[1-p\left(K-1,i\right)\right]}^{I_{\left[{y}_i=K\right]}}\right\},\end{array} $$

where ${\mathrm{I}}_{\left[{\mathrm{y}}_{\mathrm{i}}=\mathrm{w}\right]}$ is the indicator function, with value of 1 if *y*_*i*_ = *w* and *0* otherwise. Let *θ* = (*α*_1_, *α*_2_, ⋯, *α*_*K* − 1_, *β*^*T*^)^*T*^, the log-likelihood function for the proportional odds logistic model be denoted as *l*_*O*_(*θ*). The maximum likelihood estimation (MLE) $\widehat{\theta}$ for *l*_*O*_(*θ*) is usually computed using the Newton method for efficiency. The variance-covariance matrix for $\widehat{\theta}$ is estimated by $-{\left[{\partial}^2{l}_O\left(\theta \right)/\left(\partial \theta \partial {\theta}^T\right)\Big|{}_{\widehat{\theta}}\right]}^{-1}$.

Equation (1) assumes that the non-intercept model coefficients *β* remain the same for 1 ≤ *w* ≤ *K* − 1. Usually, a justification for the model assumption is needed when fitting ordinal logistic model. This assumption is called proportional odds assumption [15]. The score test is a common way to test the proportional odds assumption. To perform the score test, we first introduce the generalized ordered logit model [16], which is a generalization of the ordinal logistic model as it allows non-intercept model coefficients to be different. The generalized ordered logit model is given by

3 $$ p\left(w,i\right)=\frac{e^{\alpha_w+{\beta}_w^T{x}_i}}{1+{e}^{\alpha_w+{\beta}_w^T{x}_i}}, $$

with ${\beta}_w^T=\left({b}_{w,1},\cdots, {b}_{w,m}\right)$ for 1 ≤ *i* ≤ *n* and 1 ≤ *w* ≤ *K* − 1. Let us denote $\psi ={\left({\alpha}_1,{\beta}_1^T,\cdots, {\alpha}_2,{\beta}_{K-1}^T\right)}^T$. The log-likelihood function for this generalized model, *l*_*G*_(*ψ*)*,* is obtained by combining (3) and (2). From its definition, we see that the generalized ordered logistic model requires more parameters than the proportional odds model. Hence, model fitting for small sample size data is a big concern for the generalized ordered logistic model. To check the proportional odds assumption, we need to test whether *β*_1_ = ⋯ = *β*_*K* − 1_. As mentioned previously, suppose $\widehat{\theta}=\left\{{\widehat{\alpha}}_1,\cdots, {\widehat{\alpha}}_{K-1},\widehat{\beta}\right\}$ is the MLE for *l*_*O*_(*θ*). Let $\overset{\sim}{\psi }=\left\{{\widehat{\alpha}}_1,\widehat{\beta},\cdots, {\alpha}_{K-1},\widehat{\beta}\right\}$*.* The score test statistic is

4 $$ {T}_o={\left[\frac{\partial {l}_G\left(\psi \right)}{\partial \psi}\Big|{}_{\overset{\sim }{\psi }}\right]}^T{\left[-\frac{\partial^2{l}_G\left(\psi \right)}{\partial \psi \partial {\psi}^T}\Big|{}_{\overset{\sim }{\psi }}\right]}^{-1}\left[\frac{\partial {l}_G\left(\psi \right)}{\partial \psi}\Big|{}_{\overset{\sim }{\psi }}\right]. $$

Under the null hypothesis *β*_1_ = ⋯ = *β*_*K* − 1_, *T*_*o*_ asymptotically follows ${\chi}_{m\left(K-2\right)}^2$.

The multinomial logistic model is mainly dealing with a nominal response with unordered categories. It does not require the proportional odds assumption. Using the multinomial model on ordered data disregards the inherent information in the ordering of the response categories and is not, in general, recommended. Suppose the response variable and predictors are the same as described in the proportional odds model except that the proportional odds assumption does not hold. Let’s denote $\tilde{p}\left(w,i\right)\triangleq Pr\left(Y=w\Big|{x}_i\right)$*.* In multinomial logistic model for 1 ≤ *i* ≤ *n* and 1 ≤ *w* ≤ *K* − 1

5 $$ \tilde{p}\left(w,i\right)=\frac{e^{\alpha_w+{\beta}_w^T{x}_i}}{1+{\displaystyle {\sum}_{k=1}^{K-1}}\ {e}^{\alpha_k+{\beta}_k^T{x}_i}}. $$

The likelihood function is then given by

6 $$ L={\varPi}_{i=1}^n\left\{\tilde{p}{\left({y}_i,i\right)}^{I_{\left[{y}_i<K\right]}}{\left[1-{\displaystyle {\sum}_{k=1}^{K-1}\ \tilde{p}\left(k,i\right)}\right]}^{I_{\left[{y}_i=K\right]}}\right\}. $$

As previously mentioned *ψ*$$ =\Big({\alpha}_1,\kern0.5em {\beta}_1^T,\kern0.5em \cdots, \kern0.5em {\alpha}_{K-1}, $$$$ {\beta}_{K-1}^T\Big){}^T $$. The log-likelihood function for multinomial logistic regression is denoted as *l*_*M*_(*ψ*). The MLE $$ \widehat{\psi} $$ for multinomial logistic regression can be also obtained by the Newton method and the variance-covariance matrix for $$ \widehat{\psi} $$ is estimated by $$ -{\left[{\partial}^2{l}_M\left(\psi \right)/\left(\partial \psi \partial {\psi}^T\right)\Big|{}_{\widehat{\psi}}\right]}^{-1} $$. It is worth noting that the multinomial logistic model requires the same number of parameters as does the generalized ordered logistic model.

### Grid ordinal and multinomial logistic models

This section first proposes the grid Newton method for the MLE, which can be used for both the grid proportional odds and the multinomial logistic regression models. Then, we develop the grid proportional odds ratio test for proportional odds logistic regression.

Suppose that we want to find the MLE $\widehat{\theta}$ for the log-likelihood function *l*(*θ*) with *θ* being a column vector. We can apply the Newton method as

7 $$ {\theta}^{\left(J+1\right)}={\theta}^{(J)}-{\left[\frac{\partial^2l\left(\theta \right)}{\partial \theta \partial {\theta}^T}\Big|{}_{\theta^{(J)}}\right]}^{-1}\left[\frac{\partial l\left(\theta \right)}{\partial \theta}\Big|{}_{\theta^{(J)}}\right], $$

for *J* = 0, 1, 2, ⋯*. θ*^(*J*)^ approaches $\widehat{\theta}$ as *J* increases. Because the Newton method is very efficient, it is usually enough for *J* < 15 to achieve a tolerance 10^(−6)^ for *θ*^(*J*)^,

Suppose data are split into *U* parts in terms of observations and each part contains the same variables. Let *l*(*θ*) be the log-likelihood function for data combined from *U* parts, which can be decomposed by observations. Hence

8 $$ l\left(\theta \right)={\displaystyle {\sum}_{u=1}^U{l}_u\left(\theta \right),} $$

where *l*_*u*_(*θ*) is the log-likelihood function for data of part *u* with *u* = 1, ⋯, *U*. For the gradient and Hessian matrix of *l*(*θ*)*,* we have

9 $$ \frac{\partial l\left(\theta \right)}{\partial \theta }={\displaystyle {\sum}_{u=1}^U\frac{\partial {l}_u\left(\theta \right)}{\partial \theta }} $$

and

10 $$ \frac{\partial^2l\left(\theta \right)}{\partial \theta \partial {\theta}^T}={\displaystyle {\sum}_{u=1}^U\frac{\partial^2{l}_u\left(\theta \right)}{\partial \theta \partial {\theta}^T},} $$

respectively. We get the following grid Newton method from (9), (10) and (7)

11 $$ {\theta}^{\left(J+1\right)}={\theta}^{(J)}-{\left[{\displaystyle {\sum}_{u=1}^U\frac{\partial^2{l}_u\left(\theta \right)}{\partial \theta \partial {\theta}^T}\Big|{}_{\theta^{(J)}}}\right]}^{-1}\left[{\displaystyle {\sum}_{u=1}^U\frac{\partial {l}_u\left(\theta \right)}{\partial \theta}\Big|{}_{\theta^{(J)}}}\right]. $$

Equation (11) tells us that each Newton update can be finished by combining gradients and Hessian matrices of the partial log-likelihood functions based on corresponding sub-datasets. This equation suggests the following model fitting process in which separate datasets do not need to be pooled in the fitting process.

1. Compute gradients and Hessian matrices based on the current coefficient estimation using partial datasets separately.
2. Find overall gradients and Hessian matrices by combining the partial results obtained from Step1, then updating the coefficient estimation.

Starting from an initial value for the model coefficients, the MLE can be obtained by repeating Step 1 and Step 2 until convergence.

The above grid Newton method is used for both ordinal and multinomial logistic model coefficient estimations. The variance-covariance matrix of MLE $\widehat{\theta}$ based on the log-likelihood function *l*(*θ*) is given by $-{\left[{\partial}^2l\left(\theta \right)/\left(\partial \theta \partial {\theta}^T\right)\Big|{}_{\widehat{\theta}}\right]}^{-1}$. Using (9) and (10), we get the grid variance-covariance matrix estimates of $\widehat{\theta}$. This is a typical grid method for a variance-covariance matrix and it is suitable for both proportional odds and multinomial logistic regression. The gradients and Hessian matrices for both regression models are presented in Additional file 1.

For the grid computing for the proportional odds assumption test statistic *T*_*o*_ in (4), we first compute the grid MLE $\widehat{\theta}$ based on the log-likelihood *l*_*O*_ of ordinal logistic regression, then *T*_*o*_ is produced by using (9) and (10) to evaluate the gradient and Hessian matrix of *l*_*G*_ at $\overset{\sim}{\psi }$, where $\overset{\sim}{\psi }$ comes from the rearrangement of $\widehat{\theta}$ entries as introduced in the previous Section.

### Grid model fit assessment

Assessment of goodness-of-fit for the ordinal logistic model can be done using methods for binary logistic regression on each of *K* − 1 regressions. Additionally, Fagerland and Hosmer [17] proposed a Homer-Lemeshow type goodness-of-fit test for the proportional odds. To handle the multinomial logistic model, Hosmer and Lemeshow [9] modified several existing measures, including Pearson’s residual and R-square. Alternatively, Fagerland *et al.* [18] modified the Hosmer-Lemeshow (HL) test for the same goal. Some of these methods can be used for grid models.

We use the HL test as an example to explain grid model fit assessment. For binary logistic regression, the HL test statistic is calculated as follows. First, sorted values of the predicted probability of *Y* = 1 for all observations are split into *g* groups. *E*_*c*,*k*_ equals the sum of predicted probability of *Y* = *k* (*k* = 0, 1) in category *c*, *O*_*c*,*k*_ equals the number of observations with *Y* = *k* in category *c*. Then the test statistic is given by

$$ H{L}_b={\displaystyle \sum_{c=1}^{\mathit{\mathsf{g}}}}{\displaystyle \sum_{k=0}^1}{\left({O}_{c,k}-{E}_{c,k}\right)}^2/{E}_{c,k}, $$

which asymptotically follows ${\chi}_{g-2}^2$. In the modified statistic, the *g* groups are split based on sorted values of the predicted probability of *Y<K* for all observations. The extended HL (EHL) test statistic is defined as $H{L}_m={\displaystyle {\sum}_{c=1}^{\mathit{\mathsf{g}}}{\displaystyle {\sum}_{k=1}^K{\left({O}_{c,k}-{E}_{c,k}\right)}^2/{E}_{c,k}}}$, where *O*_*c*,*k*_ and *E*_*c*,*k*_ are defined in the same way as above. The new statistic asymptotically follows ${\chi}_{\left(g-2\right)\left(K-1\right)}^2$. *O*_*c*,*k*_ and *E*_*c*,*k*_ only requires response value and predicted probability of *Y=k* for all observations. Grid *HL*_*m*_ computing can be finished by first pooling *Y* values and corresponding predicted probability values from separate sub-datasets after grid model fitting.

### Grid Area under the ROC Curve

The rationale of grid model fitting is based on the assumption that the grid model outperforms models fitted by separate sub-datasets. However, this is not always true and actually depends on data structures. The Area Under ROC Curve (AUC) is a very popular measurement to assess model classification performance, so we propose to use the AUC to check the value of a grid model.

For ordinal logistic regression, we adopt the idea proposed by Van Calster *et al.* [12] to use the mean of *K* − 1 AUC scores for assessing the model. For the multinomial logistic regression we adopt the Hand and Till [11] AUC estimation method. For both grid models, their AUC scores can be obtained by pooling response values and predicted probabilities for necessary observations from separate sub-datasets after model fitting. To check the added value, we need to compare the grid AUC score with the AUC score for each sub-dataset.

## Results

### Simulation

The derivation of the grid method clearly implies that the grid method gives identical results as does the centralized method (i.e., the methods in which sub-datasets are pooled). Additionally, only the total sample size from all sites is important for the fitting results, and the sample size in an individual site will not affect the fitting results for a fixed total sample size. We conducted simulation studies to evaluate the accuracy of the proposed grid model estimation and to compare it with the classical centralized fitting method. Four simulation studies in different settings were performed to compare the various grid multi-category models against two corresponding centralized models. For all studies, simulated data are split into two pieces, one for model fitting, and another for AUC score evaluation and HL test. The first two studies are designed for the ordinal logistic model and the other two studies are designed for the multinomial logistic model. In Studies 1 and 2, data were simulated from an ordinal logistic model with total sample sizes 1800 and 900, respectively. In Studies 3 and 4, data were simulated from a multinomial logistic model with total sample sizes 1800 and 900, respectively. The HL tests for all binary logistic regression estimations were performed in Studies 1 and 2; the extended HL test was performed in Studies 3 and 4. In addition, an average AUC score or extended AUC score [11] was evaluated for each study.

In all studies, we simulated data so that there are 4 outcome categories (*Y* ∈ {1, 2, 3, 4}*).* For Studies 1 and 3 we used a total sample size of 1800 for centralized models and split them into 3 separate parts in three different ways: (600, 600, 600), (100, 200, 1500) and (50, 50, 1700) for the grid models. For Studies 2 and 4 we used a total sample size of 900 for centralized models and split them into 3 separate parts in three different ways: (300, 300, 300), (50, 100, 750) and (24, 26, 850) for the grid models. For all studies, each split subset was further split in half, one for model fitting and another for AUC evaluation and HL or extended HL tests. We chose two continuous covariates *x*_1_ and *x*_2_ and two binary covariates *x*_3_ and *x*_4_ (i.e., 5 coefficients for 4 covariates and intercept) in these studies. Simulation data were generated in two steps. First, we generated *x*_1_ and *x*_2_ from a standard normal distribution independently and generated *x*_3_ and *x*_4_ from a Bernoulli distribution with *p* = 0.5 independently. For Studies 1 and 2 we generated the response *y* from an ordinal distribution assuming that

$$ log\frac{Pr\left(Y\le 1\right)}{Pr\left(Y>1\right)}=-1+{x}_1+{x}_2+{x}_3+{x}_4, $$

$$ log\frac{Pr\left(Y\le 2\right)}{Pr\left(Y>2\right)}={x}_1+{x}_2+{x}_3+{x}_4, $$

and

$$ log\frac{Pr\left(Y\le 3\right)}{Pr\left(Y>3\right)}=1+{x}_1+{x}_2+{x}_3+{x}_4; $$

For Studies 3 and 4 we generated the response y from a multinomial distribution assuming that

$$ log\frac{Pr\left(Y=1\right)}{Pr\left(Y=4\right)}=2+0.5{x}_1+0.5{x}_2+0.5{x}_3+0.5{x}_4, $$

$$ log\frac{Pr\left(Y=2\right)}{Pr\left(Y=4\right)}=3+2{x}_1+2{x}_2+2{x}_3+2{x}_4, $$

and

$$ log\frac{Pr\left(Y=3\right)}{Pr\left(Y=4\right)}=1+{x}_1+{x}_2+{x}_3+{x}_4. $$

**Figure 1:**
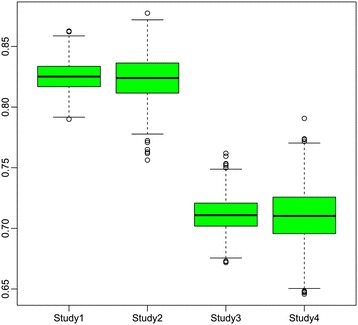
**Common box plots of AUC scores for four studies based on 1000 runs for three grid models and the corresponding centralized model.**

We conducted the simulations with 1000 runs in all studies. In Studies 1 and 2, the estimation for log odds $log\frac{Pr\left(Y\le k\right)}{Pr\left(Y>k\right)}$ equals ${\widehat{\alpha}}_k+{\widehat{\beta}}_1{x}_1+{\widehat{\beta}}_2{x}_2+{\widehat{\beta}}_3{x}_3+{\widehat{\beta}}_4{x}_4$, for *k* = 1, 2, 3. In Studies 3 and 4, the estimation for log odds $log\frac{Pr\left(Y=k\right)}{Pr\left(Y=4\right)}$ equals ${\widehat{\alpha}}_k+{\widehat{\beta}}_{1,k}{x}_1+{\widehat{\beta}}_{2,k}{x}_2+{\widehat{\beta}}_{3,k}{x}_3+{\widehat{\beta}}_{4,k}{x}_4$*,* for *k* = 1, 2, 3. Table 1 presents the results for Studies 1 and 2 and Table 2 presents the results for Studies 3 and 4. We show the average biases (Bias) and standard errors (Se) for the estimates in both tables. Table 3 provides the passing rate of the proportional odds assumption (POA) test, the HL test and both (POA&HL) tests for Studies 1 and 2. Table 3 depicts the results of the EHL test in Studies 3 and 4 among 1000 runs. Figure 1 shows the box plots of AUC scores for the four studies.

**Table 1:** **Common ordinal logistic regression estimates for three grid models with various local site sample sizes and the corresponding centralized model in Study 1 and Study 2**

		Study 1		Study 2	
	True	Bias	Se	Bias	Se
${\widehat{\alpha}}_1$	−1	−4.75e-3	1.27e-1	−1.46e-2	1.82e-1
${\widehat{\alpha}}_2$	0	−1.17e-3	1.23e-1	−3.47e-3	1.75e-1
${\widehat{\alpha}}_3$	1	1.93e-3	1.30e-1	1.02e-2	1.84e-1
${\widehat{\beta}}_1$	1	7.22e-3	8.02e-2	5.53e-3	1.14e-1
${\widehat{\beta}}_2$	1	6.60e-3	8.02e-2	3.79e-3	1.14e-1
${\widehat{\beta}}_3$	1	1.04e-2	1.42e-1	1.64e-3	2.01e-1
${\widehat{\beta}}_4$	1	7.34e-3	1.42e-1	1.36e-2	2.02e-1

**Table 2:** **Common multinomial logistic regression estimates for three grid models and the corresponding centralized model in Study 3 and Study 4**

		Study 3		Study 4	
	True	Bias	Se	Bias	Se
${\widehat{\alpha}}_1$	2	1.01e-1	4.12e-1	1.29e-1	6.01e-1
${\widehat{\beta}}_{1,1}$	0.5	3.43e-2	2.51e-1	3.76e-2	3.69e-1
${\widehat{\beta}}_{2,1}$	0.5	2.58e-2	2.51e-1	3.35e-2	3.69e-1
${\widehat{\beta}}_{3,1}$	0.5	1.39e-2	4.75e-1	1.13e-1	7.11e-1
${\widehat{\beta}}_{4,1}$	0.5	5.55e-2	4.78e-1	8.62e-2	7.08e-1
${\widehat{\alpha}}_2$	3	1.11e-1	4.09e-1	1.47e-1	5.95e-1
${\widehat{\beta}}_{1,2}$	2	5.43e-2	2.70e-1	8.40e-2	3.96e-1
${\widehat{\beta}}_{2,2}$	2	4.60e-2	2.70e-1	8.35e-2	3.95e-1
${\widehat{\beta}}_{3,2}$	2	2.55e-2	4.86e-1	1.56e-1	7.26e-1
${\widehat{\beta}}_{4,2}$	2	7.86e-2	4.89e-1	1.30e-1	7.22e-1
${\widehat{\alpha}}_3$	1	6.06e-2	4.64e-1	5.53e-2	6.80e-1
${\widehat{\beta}}_{1,3}$	1	3.23e-2	3.02e-1	5.55e-2	4.43e-1
${\widehat{\beta}}_{2,3}$	1	2.61e-2	3.02e-1	4.65e-2	4.42e-1
${\widehat{\beta}}_{3,3}$	1	1.78e-2	5.55e-1	1.18e-1	8.30e-1
${\widehat{\beta}}_{4,3}$	1	6.04e-2	5.58e-1	9.09e-2	8.25e-1

**Table 3:** **Common passing rate of the model assumption test and the model fit test in each study for three grid models and the corresponding centralized model**

	POA*	HL	POA&HL
Study 1	0.967	0.579	0.559
Study 2	0.964	0.532	0.511
		EHL	
Study 3		0.554	
Study 4		0.511	

*POA: proportional odds assumption; EHL: extended HL test.

Note that, as expected, all four studies show that the three grid methods and the corresponding centralized method produce identical results. Hence, each table or figure presents the common results for the three grid models and the corresponding centralized model.

### Two examples

In addition to simulation studies, we used two split public datasets to test our core model-fitting algorithm. The purpose was to illustrate how our core grid-fitting algorithm works. Note that these are not real multi-center studies but used for illustration purposes.

The first example is about the low birth weight dataset, which was obtained from Hosmer and Lemeshow [9] and contains 189 observations with 9 non-redundant variables. We picked 8 variables including *AGE, RACE, SMOKE, PTL, HT, UI, FTV, BWT* from the dataset, and reasonably modified several variables to create a new dataset as follows. *RACE* is a three-category variable, replaced by two binary variables: *OTHER*vs*WHITE* and *BLACK*vs*WHITE*, respectively. *PTL* is the number of premature labors with values of *0, 1*, etc., and was dichotomized into 0 and greater than 0. *FTV* is the number of physician visits, which is also dichotomized into 0 and greater than 0 as well. *BWT* is the birth weight in grams and it was categorized into 4 values (1, 2, 3 ,4) using cutoffs 3500, 3000 and 2500. *AGE*, *SMOKE*, *HT*, *UI* were kept as original, where *AGE* is continuous, *SMOKE* is binary, *HT* is binary variable for “History of hypertension", and *UI* is binary variable for “Presence of uterine irritability". We denote the new dataset as LBW.

To test the grid model fitting, we randomly picked 95 observations from LBW to create dataset LBW1 and the rest 94 observations to create LBW2. *BWT* is chosen as the 4-category response variable and the rest are covariates. Since the response is ordinal, we fitted a grid ordinal logistic model without pooling LBW1 and LBW2. Suppose the fitted value for $log\frac{Pr\left(BWT\le k\right)}{Pr\left(BWT>k\right)}$ (*k* = 1, 2, 3) is

$$ {\widehat{\alpha}}_k+{\widehat{\beta}}_1 AGE+{\widehat{\beta}}_2 OTHERvsWHITE+{\widehat{\beta}}_3 BLACKvsWHITE+{\widehat{\beta}}_4 SMOKE+{\widehat{\beta}}_5PTL+{\widehat{\beta}}_6HT+{\widehat{\beta}}_7UI+{\widehat{\beta}}_8FTV. $$

Table 4 shows the model coefficient estimates (Est) and their standard errors (Se), with z-values (Zval) equal to the ratios of Est values over according Se values and p-values (Pval) to test whether Zval is significantly different than 0.

**Table 4:** **Grid ordinal logistic model fitting by separate low birth weight datasets**

	Est	Se	Zval	Pval
${\widehat{\alpha}}_1$	−0.415	0.719	−0.578	0.562
${\widehat{\alpha}}_2$	0.828	0.722	1.147	0.251
${\widehat{\alpha}}_3$	1.807	0.730	2.473	0.013
${\widehat{\beta}}_1$	0.016	0.027	0.594	0.552
${\widehat{\beta}}_2$	−0.980	0.339	−2.891	0.003
${\widehat{\beta}}_3$	−1.245	0.424	−2.933	0.003
${\widehat{\beta}}_4$	−1.028	0.318	−3.233	0.001
${\widehat{\beta}}_5$	−0.915	0.419	−2.178	0.029
${\widehat{\beta}}_6$	−0.991	0.618	−1.605	0.108
${\widehat{\beta}}_7$	−0.972	0.402	−2.416	0.015
${\widehat{\beta}}_8$	−0.031	0.289	−0.107	0.914

The grid proportional odds assumption test was also performed and resulted in a p-value of 0.366. Hence, there is no evidence to show that the assumption for the ordinal logistic model was invalid. To justify the grid model fitting, the ordinal logistic model was also fitted for LBW1 and for LBW2, separately. Grid AUC score (GAUC), AUC score for the model fitted by LBW1 (AUC1), and AUC score for the model fitted by LBW2 (AUC2) were all evaluated by 10-fold cross validation:

$$ GAUC=0.665,AUC1=0.645,AUC2=0.568. $$

Note that in this example the data are randomly split so every subset has the same underlying population. Hence, small AUC values only result from smaller sample sizes (in subgroups). In addition, a grid HL test for grid model and HL tests for two separate models were performed using 10-fold cross validation with the same data partitions. Unfortunately, none of these models passed the HL test. This may be related to nonlinear effects of the continuous variable age, or to omitted interaction terms. However, as shown in simulation studies, failing to pass the HL test does not necessary mean the goodness-of-fit of these models are very poor.

The second example is about Mammograph experience data, which was also obtained from Hosmer and Lemeshow [9] and contains 412 observations with 6 variables. We kept the original dataset and only replaced multi-category variables by multiple binary variables. The generated new dataset was denoted as *MAM* and contained 9 variables: *ME*, *SYMPT*1, *SYMPT*2, *SYMPT*3, *PB*, *HIST*, *BSE*, *DETC*1 and *DETC*2. *ME* denotes mammograph experience with “*3 = never*", “*2 = within a year*" and “*1 = over a year ago*". Original *SYMPT* was a 4-category variable and denoted the 4 responses to “you do not need a mammograph unless you develop symptoms" from “strongly agree" to “strongly disagree". It was replaced by binary variables *SYMPT*1, *SYMPT*2 and *SYMPT*3. *PB* is a continuous variable for the degree of “perceived benefit of mammography". *HIST* is a binary variable for the response to whether “mother or sister has breast cancer history". *BSE* is the binary response to “Has anyone taught you how to examine your own breasts?". Original *DECT* was a 3-category variable and the response to “How likely is it that a mammogram could find a new case of breast cancer?". It was replaced by binary variables *DECT*1 and *DECT*2.

We first randomly picked 206 observations from *MAM* to create dataset *MAM1*, and used the remaining 206 to create *MAM2*. We used *ME* as the response. The multinomial logistic model was used to fit the dataset. We fitted a grid multinomial model without pooling *MAM1* and *MAM2*. For *k* = 1, 2, suppose the fitted value for $log\frac{Pr\left( ME=k\right)}{Pr\left( ME=3\right)}$ is

$$ {\widehat{\alpha}}_k+{\widehat{\beta}}_{1,k} SYMPT1+{\widehat{\beta}}_{2,k} SYMPT2+{\widehat{\beta}}_{3,k} SYMPT3+{\widehat{\beta}}_{4,k} PB+{\widehat{\beta}}_{5,k} HIST+{\widehat{\beta}}_{6,k}BSE+{\widehat{\beta}}_{7,k} DETC1+{\widehat{\beta}}_{8,k} DECT2 $$

Table 5 shows the model coefficient estimates (Est) and their standard errors (Se), with z-values (Zval) and p-values (Pval).

**Table 5:** **Grid multinomial logistic model fitting by separate mammography datasets**

	Est	Se	Zval	Pval
${\widehat{\alpha}}_1$	−0.986	1.111	−0.886	0.375
${\widehat{\beta}}_{1,1}$	1.132	0.547	2.067	0.038
${\widehat{\beta}}_{2,1}$	0.817	0.539	1.514	0.129
${\widehat{\beta}}_{3,1}$	−0.290	0.644	−0.450	0.652
${\widehat{\beta}}_{4,1}$	−0.148	0.076	−1.940	0.052
${\widehat{\beta}}_{5,1}$	1.065	0.459	2.319	0.020
${\widehat{\beta}}_{6,1}$	1.052	0.514	2.043	0.041
${\widehat{\beta}}_{7,1}$	−0.690	0.687	−1.004	0.314
${\widehat{\beta}}_{8,1}$	−0.924	0.713	−1.295	0.195
${\widehat{\alpha}}_2$	−2.998	1.539	−1.948	0.051
${\widehat{\beta}}_{1,2}$	2.456	0.775	3.168	0.001
${\widehat{\beta}}_{2,2}$	1.924	0.777	2.475	0.013
${\widehat{\beta}}_{3,2}$	0.110	0.922	0.119	0.905
${\widehat{\beta}}_{4,2}$	−0.219	0.075	−2.905	0.003
${\widehat{\beta}}_{5,2}$	1.366	0.437	3.122	0.001
${\widehat{\beta}}_{6,2}$	1.291	0.529	2.437	0.014
${\widehat{\beta}}_{7,2}$	0.904	1.126	0.802	0.422
${\widehat{\beta}}_{8,2}$	0.017	1.161	0.014	0.988

To justify the grid model fitting, a multinomial logistic model was also fitted for *MAM1* and for *MAM2*, separately. However, both separate models produced invalid estimates (with very large standard errors). The invalid estimates are probably due to the small number of subjects with *ME* = 2 after splitting the dataset, and the large number of parameters. This obviously shows the need for grid model fitting based on datasets *MAM1* and *MAM2* when they are not allowed to be pooled. Ten-fold cross validation was used to evaluate extended AUC score and we performed the extended HL test for the grid fitted model. The grid AUC score was 0.626 and the grid fitted model passed the extended HL test.

## Discussion

While our focus was on multi-category logit models, the grid MLE method is applicable for grid computing for various likelihood type estimation problems including other generalized linear models and generalized estimating equation models. However, when the likelihood is not separable for observations, then grid MLE may not work. For example, the Cox proportional hazards regression adopts a profile likelihood that cannot be split by observations. Hence, more effort is necessary to design a grid model for Cox proportional odds regression, which was discussed in our recent publication [19].

For the proposed grid HL test and grid AUC, *Y* values are pooled directly and not protected. To protect *Y*, the patient outcome values, we could adopt the methods proposed by Wu et al. [6] for the Grid HL test and the AUC score calculation, which avoid exchanging *Y* values. These methods are accomplished through using transmitted locally predicted probabilities and their orders. Details are given in Algorithms 1 and 2 in Wu et al. [6].

In practice, the grid model fitting using multi-site data is more complicated than what is described in this manuscript (we focused on the model fitting step). Very often, it is necessary to conduct data pre-processing before the model fitting. For example, gender may use a coding method in different sites. Hence, data harmonization is necessary before the grid model can be fitted. Another issue is missing data. One way to mitigate the problem is to deal with missing data during the pre-processing step using the same grid protocol across all sites. Another approach is to handle missing data in the grid model-fitting step, which would be cumbersome. Additionally, sometimes there are too many variables to fit the model; variable selection may thus be needed. Variable selection usually requires the construction of models and it can be incorporated into the model-fitting step. Different sites may have different variables, so choosing and harmonizing the values of common variables needs to be done before the model-fitting step. For the proposed grid models, we assumed that the data were uniformly distributed across local clinical sites, and treated the data from each local site as a random sample from the whole dataset. However, this assumption may not hold and we will consider cluster effects from different sites in our future work. We described (on page 4) that steps 1 and 2 for the grid model-fitting step need to be repeated until convergence. Each site needs to send the first derivative and Hessian matrices multiple times, which means that a reliable data transmission function is necessary for successfully fitting a grid model. Recently we produced a reliable webservice called WebGLORE for binary logistic grid fitting [20]. In our setting the data transmission was adequate but there may be settings in which this may not be the case.

## Conclusion

In the proposed grid methods, individual-level observation data were never shared during the model fitting process. This offers a practical solution for mitigating privacy issues caused by pooling all data into a central site. Grid ordinal and multinomial logistic models were introduced in detail. In terms of increasing sample sizes, grid computing is more valuable for multi-category response logistic model than it is for binary logistic regression, since the larger number of coefficient estimates in multi-category models obviously require more observations. A small sample size might result in estimations with very large bias or standard error. The ordinal logistic model was proposed to only address the ordinal response data. The multinomial logistic model is used to deal with nominal response data, which requires even more coefficients and hence more observations for proper estimation when compared to the ordinal logistic model. The theory guarantees that the proposed grid Newton method achieves accurate estimation, which is the same as the one of the classical centralized Newton method. This is consistent with simulation study results. As shown in the simulation studies, the HL test and its extension might be too strong for assessing model fit and might produce false significant test results. These are limitations for the HL test, which are discussed by Vittinghoff *et al.* [21]. Hence, other model fit assessment methods introduced by Hosmer and Lemeshow [9] could be used in addition to the extended HL test for the multinomial logistic model, and other methods for binary logistic model fit assessment could be used in addition to the HL test for the ordinal logistic model.

## Additional file

Additional file 1: **Gradients and Hessian matrices.** In this file we provide the gradients and the Hessian matrices for all log-likelihood functions used in this manuscript.
