# Supplementary material for: Grid multi-category response logistic models
Source: BMC Med Inform Decis Mak. 2015 Feb 18;15:10. doi: 10.1186/s12911-015-0133-y (PMC4342889; doi:10.1186/s12911-015-0133-y)
Supplement: Additional file 1: — Gradients and Hessian matrices. In this file we provide the gradients and the Hessian matrices for all log-likelihood functions used in this manuscript. [file 12911_2015_133_MOESM1_ESM.pdf]

Gradients and Hessian matrices

Computing the gradient and the Hessian matrix of a log-likelihood is the key point for computing both centralized and grid MLE. Here are the gradients and the Hessian matrices for all log-likelihood functions used in this manuscript.

Ordinal logistic model

Based on (2), the log-likelihood function is given by

$$l_O(\theta)=\sum_{i=1}^{n}\left\{I_{[y_i=1]}log\frac{e^{\alpha_1+\beta^T x_i}}{1+e^{\alpha_1+\beta^T x_i}}+\sum_{w=2}^{K-1}I_{[y_1=w]}log\left[\frac{e^{\alpha_w+\beta^T x_i}}{1+e^{\alpha_w+\beta^T x_i}}-\frac{e^{\alpha_{w-1}+\beta^T x_i}}{1+e^{\alpha_{w-1}+\beta^T x_i}}\right]+I_{[y_i=K]}log\left[1-\frac{e^{\alpha_{w-1}+\beta^T x_i}}{1+e^{\alpha_{w-1}+\beta^T x_i}}\right]\right\}.$$

Then we get the components of the gradient of $l_O(\theta)$ as:

$$\frac{\partial l_O(\theta)}{\partial\alpha_1}=\sum_{i=1}^{n}\left\{I_{[y_i=1]}\left[1-\frac{e^{\alpha_1+\beta^T x_i}}{1+e^{\alpha_1+\beta^T x_i}}\right]+I_{[y_i=2]}\left[-\frac{e^{\alpha_1}}{e^{\alpha_2}-e^{\alpha_1}}-\frac{e^{\alpha_1+\beta^T x_i}}{1+e^{\alpha_1+\beta^T x_i}}\right]\right\},$$

for $2\le w\le K-2$

$$\frac{\partial l_O(\theta)}{\partial\alpha_w}=\sum_{i=1}^{n}\left\{I_{[y_i=w]}\left[\frac{e^{\alpha_w}}{e^{\alpha_w}-e^{\alpha_{w-1}}}-\frac{e^{\alpha_w+\beta^T x_i}}{1+e^{\alpha_w+\beta^T x_i}}\right]+I_{[y_i=w+1]}\left[-\frac{e^{\alpha_w}}{e^{\alpha_{w+1}}-e^{\alpha_w}}-\frac{e^{\alpha_w+\beta^T x_i}}{1+e^{\alpha_w+\beta^T x_i}}\right]\right\},$$

$$\frac{\partial l_O(\theta)}{\partial\alpha_{K-1}}=\sum_{i=1}^{n}\left\{I_{[y_i=K-1]}\left[\frac{e^{\alpha_{K-1}}}{e^{\alpha_{K-1}}-e^{\alpha_{K-2}}}-\frac{e^{\alpha_{K-1}+\beta^T x_i}}{1+e^{\alpha_{K-1}+\beta^T x_i}}\right]+I_{[y_i=K]}\left[-\frac{e^{\alpha_{K-1}+\beta^T x_i}}{1+e^{\alpha_{K-1}+\beta^T x_i}}\right]\right\},$$

and

$$\frac{\partial l_O(\theta)}{\partial\beta}=\sum_{i=1}^{n}\left\{I_{[y_i=1]}\left[\left(1-\frac{e^{\alpha_1+\beta^T x_i}}{1+e^{\alpha_1+\beta^T x_i}}\right)x_i\right]+\sum_{w=2}^{K-1}I_{[y_i=w]}\left[\left(1-\frac{e^{\alpha_w+\beta^T x_i}}{1+e^{\alpha_w+\beta^T x_i}}-\frac{e^{\alpha_{w-1}+\beta^T x_i}}{1+e^{\alpha_{w-1}+\beta^T x_i}}\right)x_i\right]+I_{[y_i=K]}\left[\left(-\frac{e^{\alpha_{K-1}+\beta^T x_i}}{1+e^{\alpha_{K-1}+\beta^T x_i}}\right)x_i\right]\right\}.$$

Next the entries of the Hessian matrix of $l_O(\theta)$ are given by

$$\frac{\partial^2 l_O(\theta)}{\partial\alpha_1^2}=\sum_{i=1}^{n}\left\{I_{[y_i=1]}\left[-\frac{e^{\alpha_1+\beta^T x_i}}{(1+e^{\alpha_1+\beta^T x_i})^2}\right]+I_{[y_i=2]}\left[-\frac{e^{\alpha_1}e^{\alpha_2}}{(e^{\alpha_2}-e^{\alpha_1})^2}-\frac{e^{\alpha_1+\beta^T x_i}}{(1+e^{\alpha_1+\beta^T x_i})^2}\right]\right\},$$

for $2\le w\le K-2$

$$\frac{\partial^2 l_O(\theta)}{\partial\alpha_w^2}=\sum_{i=1}^{n}\left\{I_{[y_i=w]}\left[-\frac{e^{\alpha_{w-1}}e^{\alpha_w}}{(e^{\alpha_w}-e^{\alpha_{w-1}})^2}-\frac{e^{\alpha_w+\beta^T x_i}}{(1+e^{\alpha_w+\beta^T x_i})^2}\right]+I_{[y_i=w+1]}\left[-\frac{e^{\alpha_w}e^{\alpha_{w+1}}}{(e^{\alpha_{w+1}}-e^{\alpha_w})^2}-\frac{e^{\alpha_w+\beta^T x_i}}{(1+e^{\alpha_w+\beta^T x_i})^2}\right]\right\},$$

for $1\le w\le K-2$

$$\frac{\partial^2 l_O(\theta)}{\partial\alpha_w\,\partial\alpha_{w+1}}=\sum_{i=1}^{n}\left\{I_{[y_i=w+1]}\left[\frac{e^{\alpha_w}e^{\alpha_{w+1}}}{(e^{\alpha_{w+1}}-e^{\alpha_w})^2}\right]\right\},$$

$$\frac{\partial^2 l_O(\theta)}{\partial\alpha_{K-1}^2}=\sum_{i=1}^{n}\left\{I_{[y_i=K-1]}\left[-\frac{e^{\alpha_{K-2}}e^{\alpha_{K-1}}}{(e^{\alpha_{K-1}}-e^{\alpha_{K-2}})^2}-\frac{e^{\alpha_{K-1}+\beta^T x_i}}{(1+e^{\alpha_{K-1}+\beta^T x_i})^2}\right]+I_{[y_i=K]}\left[-\frac{e^{\alpha_{K-1}+\beta^T x_i}}{(1+e^{\alpha_{K-1}+\beta^T x_i})^2}\right]\right\},$$

for1 $\le w\le K-1$

$$\frac{\partial^2 l_O(\theta)}{\partial\alpha_w\,\partial\beta} = \sum_{i=1}^{n} \left\{ (I_{[y_i=w]} + I_{[y_i=w+1]}) \left[-\frac{e^{\alpha_w+\beta^T x_i}}{(1+e^{\alpha_w+\beta^T x_i})^2} \right] x_i \right\},$$

and

$$\frac{\partial^2 l_O(\theta)}{\partial\beta\,\partial\beta^T} = X^T diag\{d_1, \cdots, d_n\} X$$

with

$$d_i = I_{[y_i=1]} \left[-\frac{e^{\alpha_1+\beta^T x_i}}{(1+e^{\alpha_1+\beta^T x_i})^2} \right] + \sum_{w=2}^{K-1} I_{[y_i=w]} \left[-\frac{e^{\alpha_w+\beta^T x_i}}{(1+e^{\alpha_w+\beta^T x_i})^2} - \frac{e^{\alpha_w+\beta^T x_i}}{(1+e^{\alpha_w+\beta^T x_i})^2} \right] + I_{[y_i=K]} \left[-\frac{e^{\alpha_{K-1}+\beta^T x_i}}{(1+e^{\alpha_{K-1}+\beta^T x_i})^2} \right].$$

<u>Generalized ordered logit model</u>

To allow a more compact notation, by assuming that the x_i's contain intercept 1 we rewrite the generalized ordered logit model (3) as

$$p(w,i) = \frac{e^{\psi_w^T x_i}}{1+e^{\psi_w^T x_i}}.$$

Then for $\psi = (\psi_1^T, \cdots, \psi_{K-1}^T)^T$ (2) produces the following log-likelihood function:

$$l_G(\psi) = \sum_{i=1}^{n} \left\{ I_{[y_i=1]} \log \frac{e^{\psi_1^T x_i}}{1+e^{\psi_1^T x_i}} + \sum_{w=2}^{K-1} I_{[y_1=w]} \log \left[\frac{e^{\psi_w^T x_i}}{1+e^{\psi_w^T x_i}} - \frac{e^{\psi_{w-1}^T x_i}}{1+e^{\psi_{w-1}^T x_i}} \right] + I_{[y_i=K]} \log \left[1 - \frac{e^{\psi_{w-1}^T x_i}}{1+e^{\psi_{w-1}^T x_i}} \right] \right\}.$$

The components of the gradient of the log-likelihood above are given by

$$\frac{\partial l_G(\psi)}{\partial\psi_1} = \sum_{i=1}^{n} \left\{ I_{[y_i=1]} \left[1 - \frac{e^{\psi_1^T x_i}}{1+e^{\psi_1^T x_i}} \right] x_i + I_{[y_i=2]} \left[-\frac{e^{\psi_1^T x_i}}{e^{\psi_2^T x_i} - e^{\psi_1^T x_i}} - \frac{e^{\psi_1^T x_i}}{1+e^{\psi_1^T x_i}} \right] x_i \right\},$$

for $2 \le w \le K-2$

$$\frac{\partial l_G(\psi)}{\partial\psi_w} = \sum_{i=1}^{n} \left\{ I_{[y_i=w]} \left[\frac{e^{\psi_w^T x_i}}{e^{\psi_w^T x_i} - e^{\psi_{w-1}^T x_i}} - \frac{e^{\psi_w^T x_i}}{1+e^{\psi_w^T x_i}} \right] x_i + I_{[y_i=w+1]} \left[-\frac{e^{\psi_w^T x_i}}{e^{\psi_{w+1}^T x_i} - e^{\psi_w^T x_i}} - \frac{e^{\psi_w^T x_i}}{1+e^{\psi_w^T x_i}} \right] x_i \right\},$$

$$\frac{\partial l_G(\psi)}{\partial\psi_{K-1}} = \sum_{i=1}^{n} \left\{ I_{[y_i=K-1]} \left[\frac{e^{\psi_{K-1}^T x_i}}{e^{\psi_{K-1}^T x_i} - e^{\psi_{K-2}^T x_i}} - \frac{e^{\psi_{K-1}^T x_i}}{1+e^{\psi_{K-1}^T x_i}} \right] x_i + I_{[y_i=K]} \left[-\frac{e^{\psi_{K-1}^T x_i}}{1+e^{\psi_{K-1}^T x_i}} \right] x_i \right\}.$$

Next the entries of the Hessian matrix of $l_O(\theta)$ are given by

$$\frac{\partial^2 l_G(\psi)}{\partial\psi_1\,\partial\psi_1^T} = X^T diag\{d_{1,1}, \cdots, d_{1,n}\} X$$

with

$$d_{1,i} = I_{[y_i=1]} \left[-\frac{e^{\psi_1^T x_i}}{(1+e^{\psi_1^T x_i})^2} \right] + I_{[y_i=2]} \left[-\frac{e^{\psi_1^T x_i} e^{\psi_2^T x_i}}{(e^{\psi_2^T x_i} - e^{\psi_1^T x_i})^2} - \frac{e^{\psi_1^T x_i}}{(1+e^{\psi_1^T x_i})^2} \right],$$

for $2 \le w \le K-2$

$$\frac{\partial^2 l_G(\psi)}{\partial\psi_w\,\partial\psi_w^T} = X^T diag\{d_{w,1}, \cdots, d_{w,n}\} X$$

with

$$d_{w,i} = I_{[y_i=w]} \left[-\frac{e^{\psi_{w-1}^T x_i} e^{\psi_w^T x_i}}{(e^{\psi_w^T x_i} - e^{\psi_{w-1}^T x_i})^2} - \frac{e^{\psi_w^T x_i}}{(1+e^{\psi_w^T x_i})^2} \right] + I_{[y_i=w+1]} \left[-\frac{e^{\psi_w^T x_i} e^{\psi_{w+1}^T x_i}}{(e^{\psi_{w+1}^T x_i} - e^{\psi_w^T x_i})^2} - \frac{e^{\psi_w^T x_i}}{(1+e^{\psi_w^T x_i})^2} \right],$$

$$\frac{\partial^2 l_G(\psi)}{\partial\psi_{K-1}\,\partial\psi_{K-1}^T} = X^T diag\{d_{K-1,1}, \cdots, d_{K-1,n}\} X$$

with

$$d_{K-1,i} = I_{[y_i=K-1]}\left[-\frac{e^{\psi_{K-2}^T x_i} e^{\psi_{K-1}^T x_i}}{(e^{\psi_{K-1}^T x_i} - e^{\psi_{K-2}^T x_i})^2} - \frac{e^{\psi_{K-1}^T x_i}}{(1+e^{\psi_{K-1}^T x_i})^2}\right] + I_{[y_i=K]}\left[-\frac{e^{\psi_{K-1}^T x_i}}{(1+e^{\psi_{K-1}^T x_i})^2}\right],$$

and for $1 \le w \le K-2$

$$\frac{\partial^2 l_G(\psi)}{\partial\psi_{K-1}\,\partial\psi_{w+1}^T} = X^T diag\{d_{w,w+1,1},\cdots,d_{w,w+1,n}\}X$$

with

$$d_{w,w+1,i} = I_{[y_i=w+1]}\left[\frac{e^{\psi_w^T x_i} e^{\psi_{w+1}^T x_i}}{(e^{\psi_{w+1}^T x_i} - e^{\psi_w^T x_i})^2}\right]$$

<u>Multinomial logistic model</u>

As in last section, by assuming that the x_i's contain intercept 1, we rewrite the multinomial logistic model (5) as

$$\tilde{p}(w,i) = \frac{e^{\psi_w^T x_i}}{1+\sum_{k=1}^{K-1} e^{\psi_k^T x_i}}.$$

Then for $\psi = (\psi_1^T,\cdots,\psi_{K-1}^T)^T$ (6) produces the following log-likelihood function:

$$l_M(\psi) = \sum_{i=1}^{n} I_{[y_i \le K]}\psi_{y_i}^T x_i - \log\left[1+\sum_{w=1}^{K-1} e^{\psi_w^T x_i}\right].$$

Then for $1 \le w \le K-1$ the components of the gradient of the log-likelihood function are given by

$$\frac{\partial l_M(\psi)}{\partial\psi_w} = \sum_{i=1}^{n}\left[I_{[y_i=w]} - \frac{e^{\psi_w^T x_i}}{1+\sum_{v=1}^{K-1} e^{\psi_v^T x_i}}\right]x_i.$$

And for $1 \le w_1 \le K-1$ and $1 \le w_2 \le K-1$ the entries of the Hessian matrix of the log-likelihood function are given by

$$\frac{\partial^2 l_M(\psi)}{\partial\psi_{w_1}\,\partial\psi_{w_2}^T} = X^T diag\{d_{w_1,w_2,1},\cdots,d_{w_1,w_2,n}\}X$$

with

$$d_{w_1,w_2,i} = \left[\frac{e^{\psi_{w_1}^T x_i}}{1+\sum_{v=1}^{K-1} e^{\psi_v^T x_i}} - I_{[w_1=w_2]}\right]\frac{e^{\psi_{w_2}^T x_i}}{1+\sum_{v=1}^{K-1} e^{\psi_v^T x_i}}.$$
